# Platform for the interdisciplinary study of cardiovascular, metabolic and neurovascular diseases (PICMAN) protocol

**DOI:** 10.1038/s41598-023-47407-y

**Published:** 2023-11-22

**Authors:** Mayank Dalakoti, Melvin Khee Shing Leow, Chin Meng Khoo, Hayang Yang, Lieng Hsi Ling, Mark Muthiah, Eunice Tan, Jonathan Lee, Yock Young Dan, Nicholas Chew, Wei Qiang Seow, Poh Loong Soong, Louis Gan, Rijan Gurung, Matthew Ackers-Johnson, Han Wei Hou, Karishma Sachaphibulkij, Paul MacAry, Gwen Low, Christy Ang, Tee Joo Yeo, Andie Hartanto Djohan, Tony Li, Wesley Yeung, Rodney Soh, Ching Hui Sia, Vinay Panday, Shaun S. E. Loong, Benjamin Y. Q. Tan, Leonard L. L. Yeo, Lynette Teo, Pierce Chow, Roger Foo

**Affiliations:** 1 Department of Cardiology, National University Heart Centre, Singapore, Singapore; 2 Department of Medicine, Ng Teng Fong General Hospital, Singapore, Singapore; 3 Department of Medicine, National University Health System, Singapore, Singapore; 4 Yong Loo Lin School of Medicine, National University of Singapore, Singapore, Singapore; 5 Center for Life Sciences, National University of Singapore-Cambridge Cell Phenotyping Centre, Singapore, Singapore; 6 Lee Kong Chian School of Medicine, Nanyang Technological University, Singapore, Singapore; 7 National University of Singapore Cardiovascular Disease Translational Research Program, Singapore, Singapore; 8 Duke-NUS Medical School, Singapore, Singapore; 9 Department of Radiology, National University Health System, Singapore, Singapore

**Keywords:** Cardiovascular biology, Predictive markers, Metabolic syndrome, Clinical trials, Translational research, Risk factors, Clinical genetics, Inflammation, Molecular medicine

## Abstract

Through extensive multisystem phenotyping, the central aim of Project PICMAN is to correlate metabolic flexibility to measures of cardiometabolic health, including myocardial diastolic dysfunction, coronary and cerebral atherosclerosis, body fat distribution and severity of non-alcoholic fatty liver disease. This cohort will form the basis of larger interventional trials targeting metabolic inflexibility in the prevention of cardiovascular disease. Participants aged 21–72 years with no prior manifest atherosclerotic cardiovascular disease (ASCVD) are being recruited from a preventive cardiology clinic and an existing cohort of non-alcoholic fatty liver disease (NAFLD) in an academic medical centre. A total of 120 patients will be recruited in the pilot phase of this study and followed up for 5 years. Those with 10-year ASCVD risk ≥ 5% as per the QRISK3 calculator are eligible. Those with established diabetes mellitus are excluded. Participants recruited undergo a detailed assessment of health behaviours and physical measurements. Participants also undergo a series of multimodality clinical phenotyping comprising cardiac tests, vascular assessments, metabolic tests, liver and neurovascular testing. Blood samples are also being collected and banked for plasma biomarkers, ‘multi-omics analyses’ and for generation of induced pluripotent stem cells (iPSC). Extensive evidence points to metabolic dysregulation as an early precursor of cardiovascular disease, particularly in Asia. We hypothesise that quantifiable metabolic inflexibility may be representative of an individual in his/her silent, but high-risk progression towards insulin resistance, diabetes and cardiovascular disease. The platform for interdisciplinary cardiovascular-metabolic-neurovascular diseases (PICMAN) is a pilot, prospective, multi-ethnic cohort study.

## Introduction

Collectively unified by high insulin resistance—diabetes, pre-diabetes and metabolic syndrome are highly prevalent and strongly antecedent to cardiovascular morbidity and mortality in Singapore. According to recent projections, over 1 million Singaporeans will have diabetes by 2050, if nothing is done to avert the trajectory^1^. Non-alcoholic fatty liver disease (NAFLD) is a silent manifestation of metabolic syndrome and is also associated with insulin resistance and cardiovascular disease. Southeast Asia has one of the highest prevalence of NAFLD and its prevalence is expected to grow in Asia^2–4^. Similar to dysregulated adipokine secretion from inflamed adipose tissues in people with insulin resistance, people with NAFLD may also express hepatokines in the circulation thereby promoting systemic inflammation and resulting in increased cardiometabolic risks^5^. This rapidly growing prevalence of NAFLD will with time, cause increase in end stage liver diseases and cardiovascular disease, cancer, diabetes and obesity^6^.

Overall, although the progression of metabolic syndrome and diabetes are correlated with microvascular and macrovascular disease, inflammation, oxidative stress and hypercoagulability, an often neglected and under-recognised component is metabolic flexibility^7^. Multiple lines of evidence suggest that metabolic inflexibility predates overt metabolic syndrome and its deterioration with increasing insulin resistance heralds the onset of diabetes and CVD^8^. Metabolic flexibility describes the body’s efficiency to switch its utilisation of different fuel substrates to match nutrient availability and while transitioning between fasting and fed states in response to changes in energy demand. Characteristically, the shift between glucose and fat oxidation, reflects the mitochondrial preferential switch between alternative fuel sources. Conversely, metabolic inflexibility is characterised by nutrient overload, heightened substrate competition, leading to mitochondrial indecision, impaired fuel switching and energy dysregulation. Even in the myocardium during heart failure, there is a shift from fatty acid oxidation to glucose usage, increasing ketone body oxidation and branched chain amino acid catabolism, but whether these mechanisms are adaptive and compensatory, or deleterious and therefore therapeutic targets, remains controversial and unresolved^7, 8^. Similarly, whether or how reversal of myocardial metabolic inflexibility is attainable, and whether that relates to a reversal in disease trajectory, remains to be proven. Nonetheless, changes in metabolism and mitochondrial function appear to precede cardiac dysfunction, with much evidence supporting metabolic dysregulation as one of the earliest precursors of cardiovascular disease. Most pertinently, questions remain about how the utilisation of multiple energy sources in the heart is regulated, what determines the balance in patients with metabolic inflexibility, how myocardial metabolic flexibility correlate with systemic metabolic flexibility, and how these measures correlate with the progression of cardiometabolic disease. Indeed, if impaired metabolic flexibility precedes overt metabolic disease or adverse cardiovascular outcomes, we anticipate that this may be how to discern individuals at the highest risk. An important objective of our project is to establish what the broad range of normal to impaired metabolic flexibility is in a cohort of local patients. The total quantity of adipose tissues and the pattern of distribution in the body has been established to correlate with insulin resistance, through both preclinical models and human data^9^. Adipocytes have been found to be not simply inert storage vessels of lipid cargoes, but active adipokines secreters, many of which are proinflammatory and antagonistic to the action of insulin. In the recent decade, advanced imaging and phenotyping had revealed that the significance of the location of fat depots as an important factor to metabolic health. Subcutaneous fat has been found to have more favourable metabolic features whereas visceral fat is more pathogenic by comparison as it has a pronounced proinflammatory adipokine profile and strongly linked to other metabolic risk factors such as insulin resistance^10, 11^. With greater insulin resistance, there is a higher risk of metabolic syndrome, including conditions such as dyslipidaemia and hypertension, both of which drive the risk of CVD upwards. Clinically, it has been shown that there was a more than two-fold increased risk of cardiovascular mortality in individuals with lower BMI with obese waist circumference, as compared to individuals with NAFLD and overweight BMI with a healthy waist circumference^12^. Hence, adipose tissue distribution has been established to play a role in CVD. Henceforth through this study, we aim to further study the association of metabolic inflexibility with measures of body fat distribution.

Another key research interest in this study and cardiometabolic disease is elucidating the role of the gut microbiome in cardiovascular disease (CVD). The discovery of notable microbial species has been found to associate with the development of CVD and other metabolic diseases such as diabetes. Research findings continuously provide additional support for the presence of shared microbial-mediated mechanisms that impact metabolic syndrome, diabetes, and the risk of CVD^13^. For example, gut microbiota has been identified to be involved in the regulation of blood pressure through the modulation of the TH17 axis, among other mechanisms^14–16^. In addition, for conditions such as atherosclerosis and endothelial dysfunction, some strains of microorganisms have been identified to be present in the plaques^17^, likely present through translocation from the gut. Various metabolic processes have been found to mediate the pathogenic effects of a suboptimal microbiota, where one key example is the trimethylamine (TMA)/trimethylamine N-oxide (TMAO) and bile acid pathways, which has been identified to be associated with an increased risk of CVD^18^. While studies have established the association of the gut microbiota with some CVD, evidence for the role of the gut microbiota in metabolic flexibility remains to be fully characterised^19^. This leads to one of the objectives of our study, to characterise the gut microbiome landscape in individuals with a spectrum of cardiovascular-metabolic function, which hopes to help us uncover microbial mechanisms with both diagnostic and therapeutic potential.

There are currently limited means which convincingly modify or improve metabolic flexibility. Lifestyle interventions including exercise, calorie restriction, time-restricted feeding have been proposed to reverse metabolic inflexibility, and therefore avert cardiovascular outcomes. Other means may be through medications such as the newly approved sodium glucose co-transporter 2 (SGLT2) inhibitors^20^. More information is urgently needed to correlate metabolic flexibility to cardiovascular function, extend its association to the presence or absence of insulin resistance or diabetes, and to enable further studies on interventions.

On their own, cardiovascular function and metabolic assessment of NAFLD/NASH patients have been carried out elsewhere before^21^. Similarly, metabolic assessment of patients with cardiovascular disease, or even diabetes, has also been studied^22^. The careful triangulation of cardiovascular and metabolic assessment of patients together with liver fibrosis assessment, lends a unique and attractive opportunity using layers of multiple imaging and non-imaging-based biomarkers, to map out the full range of normal to abnormal cardiovascular-metabolic function and associations. We will plot these on the backdrop of the spectrum of fatty liver disease, whilst the non-fatty liver disease control cohort will be used to benchmark the cardiovascular and metabolic phenotype in cross-section using a community living cohort free of cardiovascular events. This pilot study will lay the foundation to kick-start larger detailed studies to discover novel surrogate markers for impaired metabolic flexibility, opportunities to study new therapeutics that target metabolic flexibility, predating overt disease, and tackling preventive cardiology. The ultimate goal is to be able to steer our population away from the spiralling progression towards insulin resistance, diabetes, and ultimately adverse cardiovascular outcomes.

## Methods/design

**Figure 1:**
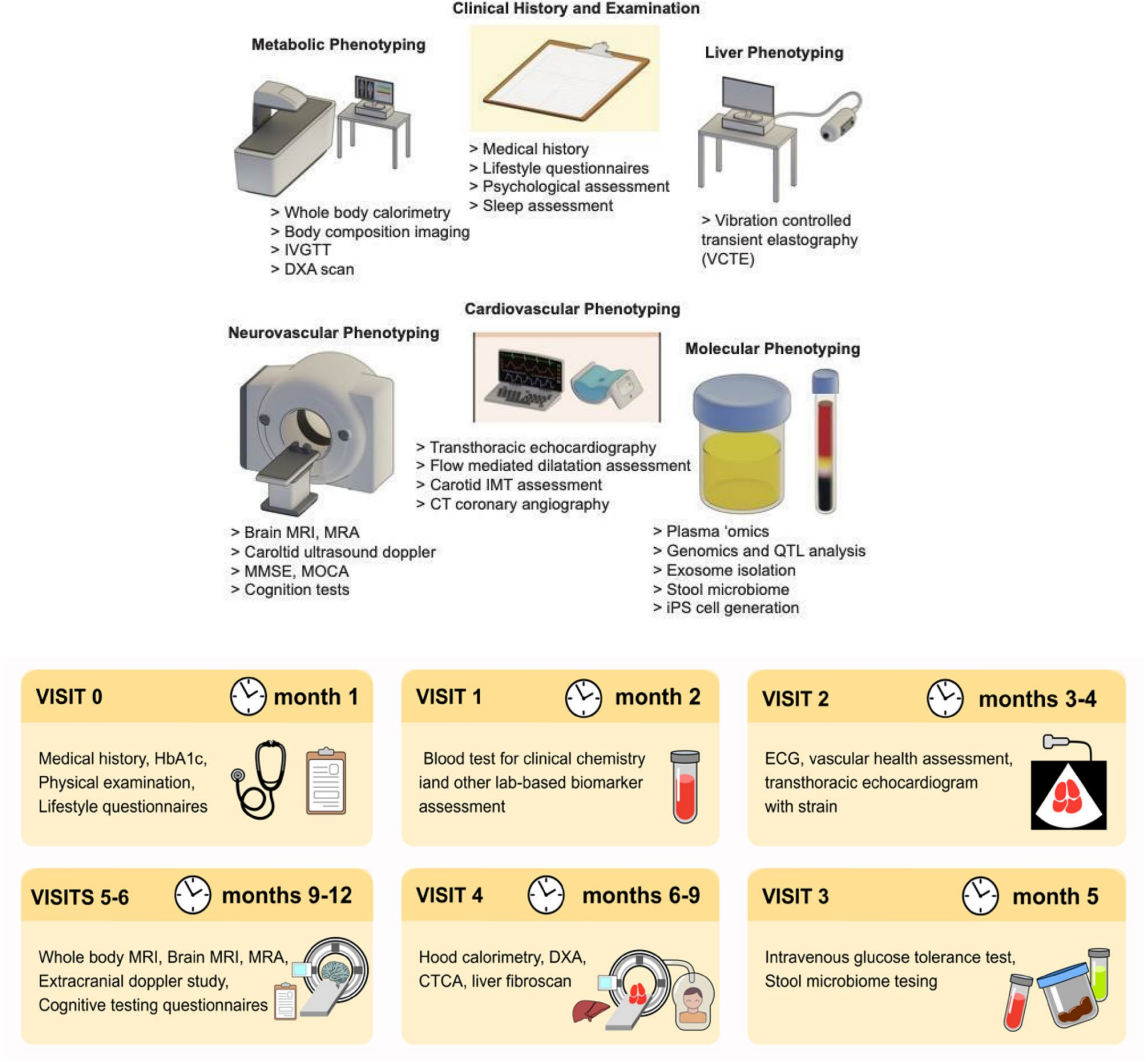
(Upper panel) Assessments and phenotyping performed; (lower panel) Timepoints of assessments and phenotyping.

We will study 120 individuals consisting of patients with (a) NAFLD and (b) no NAFLD, with a 10-year ASCVD risk > 5%^23^. The NAFLD cohort has been recruited and a subset is recalled and channelled towards this study. Individuals from this cohort were recruited from the hepatology clinic for clinical indications for liver biopsy at the request of their hepatologist. The individuals have undergone liver biopsy as a confirmatory and staging test for NAFLD^24^. Table 1 provides an overview of the breakdown of distinct NAFLD histological subtypes within this cohort. The second group is recruited from the cardiac clinics and primary care clinics. Table 2 provides an overview of the recruitment plan. Figure 1 outlines the assessment and phenotyping performed.

**Table 1:** Breakdown of the biopsy proven and staged NAFLD in the NAFLD cohort.

NAFLD subtype	N
Simple steatosis (NAFLD without NASH)	28
NASH without significant fibrosis (F0-1)	28
NASH without advanced fibrosis (F2)	17
NASH with advanced fibrosis (F3-4)	44
Burnt out cirrhosis	5
Total	122

**Table 2:** Recruitment of PICMAN cohort.

PICMAN	NAFLD cohort, N = 60	Cardiovascular cohort N = 60
Liver phenotyping	Done	To be performed
CV phenotyping	To be performed	To be performed
Metabolic phenotyping	To be performed	To be performed

### Aims

1. To define norms for metabolic flexibility in a multi-ethnic Southeast Asian cohort of individuals without manifest ASCVD.
2. To map the association of metabolic inflexibility with cardiovascular measures of diastolic dysfunction and coronary and cerebral atherosclerosis.
3. To map the association of metabolic inflexibility with measures of body fat distribution.
4. To map the correlation between metabolic flexibility and insulin sensitivity along the spectrum of subclinical cardiovascular disease and severity of non-alcoholic fatty liver disease.
5. To map the associations of the gut microbiome as well as metabolomic and molecular markers with cardio-cerebrovascular measures, metabolic flexibility and liver fibrosis.

### Eligibility and recruitment

Participants between the ages of 21 and 72 at screening without a prior history of MACE, coronary artery disease diagnosis, established diabetes, and 10-year ASCVD risk > 5% based on QRISK3 calculator are eligible for PICMAN (Table 3)^23^. All participants would provide informed consent to be included. Recruitment of individuals takes place at NUHS, where community-dwelling individuals without cardiac disease will be referred via outreach to primary care and community partners in Singapore. Our inclusion and exclusion criteria are intended to offer inclusion of individuals not characteristically “high risk” for cardiovascular and metabolic disease. At 5% risk, this marks the traditional cut-off between low and elevated risk. Individuals with diabetes mellitus have been found to have a higher incidence of cardiovascular disease, making diabetes a cardiovascular disease equivalent. As such, we have chosen only to include those with pre-diabetes without manifest diabetes, as defined by a HbA1c < 6.5% at initial screening with no prior diagnosis of diabetes by a physician.

**Table 3:** PICMAN Inclusion/exclusion criteria.

Inclusion criteria	Patients aged 21–72 at screening 10-year ASCVD risk > 5% measured by QRISK 3 calculator^23^
Exclusion criteria	Established DM (Prior diagnosis, long term medications or HbA1c ≥ 6.5) Established CVD, such as ischemic heart disease, cerebrovascular accident, heart failure, atrial fibrillation Pregnancy Known contraindications to CT scan, such as allergy to contrast

### Clinical assessments

All participants will undergo medical history review, extensive lifestyle questionnaires encompassing diet, physical activity, stress management and sleep.

### Metabolic phenotyping

Participants will undergo an extensive metabolic phenotyping assessment. These comprise (a) indirect whole-body calorimetry (WBC), (b) body composition imaging, (c) Intravenous glucose tolerance test (IVGTT) and (d) Dual Energy X- ray Absorptiometry (DXA) scan.

- Indirect calorimetry. An early hallmark of insulin resistance is metabolic inflexibility^25^, referring to blunted metabolic response upon fuel substrate changes^10^. Healthy transitions from fasting to feeding requires efficient systemic insulin-dependent shifts from fatty acid to glucose oxidation. With insulin resistance and beta cell dysfunction, the insulin-dependent switch is blunted, hence metabolic inflexibility. Resting energy expenditure (REE) and respiratory quotient (RQ) will be measured by hood calorimetry with a predefined liquid mixed meal challenge. Metabolic flexibility is calculated by a change in respiratory quotient (ΔRQ) as measured by the difference in RQ between the fasting state and after a liquid mixed meal challenge. Protocol for the mixed meal challenge was developed based on a systematic review defining an optimal nutritional stress challenge^26^.The liquid diet adopted for our PICMAN study is using a proprietary formulation of Ensure liquid meal marketed by Abbott company, each serving size of 200 mL comprising 30% fat (= 6.6 g) (10% saturated fat, 10% PUFA, 10% MUFA), 15% protein (= 7.4 g) and 55% carbohydrates (= 26 g), totaling 200 kcal per 200 mL. Participants consume 400 mls of Ensure^R^ Plus that provides 600 kcal of energy, 25 g protein, 19.7 g fat, 80.8 g carbohydrate.
- Body composition imaging. Participants will undergo body composition imaging via the AMRA scan^27^. This is a full body MRI, which will provide outputs of compartmental adipose tissue quantification (visceral fat volume, subcutaneous fat volume, muscle fat infiltration percentage, liver fat percentage), as well as muscle measurements (total thigh volume, anterior and posterior volumes of left and right thighs respectively).
- Intravenous glucose tolerance test. The standard IVGTT protocol was adopted according to Bergman’s minimal model. This model uses an iv bolus of 50% dextrose^28^. The absolute amount of dextrose infused differs from subject to subject, being dependent on body weight according to the dosing of 0.3 g/kg body weight. The bolus is administered rapidly over a duration of 1 min in the IVGTT protocol, with the total duration of blood sampling that follows would take place over 3 h. By challenging the body with a rapid IV bolus of concentrated 50% glucose, the insulin response and glucokinetics during the IVGTT will reveal the underlying physiology of glucose homeostasis to a more detailed degree. Insulin sensitivity is a parameter denoted by ‘Si’ and is a readout of the MINMOD Millenium program. The MINMOD analysis takes place after measured plasma glucose and insulin levels are gathered at the specified time points as per IVGTT protocol. The mathematical formulation of the minimal model yields the equation: Si = a2/a1, the ratio of the coefficients of glucose disappearance (from glucose uptake into insulin-sensitive tissues) and plasma insulin concentration respectively. When compared against the gold standard yardstick of insulin sensitivity, the hyperinsulinemic euglycemic glucose clamp, this equates to the glucose infusion rate that clamps the plasma glucose to euglycemia under continuous insulin infusion, which is the definition of insulin sensitivity^29^. Insulin sensitivity (= Si) values are henceforth derived when the glucose and insulin data are fitted into the minimal model of Bergman’s MinMod Millenium software. Insulin resistance is not only important as a risk determinant for developing diabetes, over the long term, insulin resistance also correlates to cardiovascular risk and disease outcomes^30^.
- Dual energy X- ray absorptiometry. These will measure fat, muscle and bone mass based on the differential absorption by body tissues of X-ray photons of 2 energy levels. It will provide outputs of total body fat percentage (as a percent of the body that is composed of fat), fat mass index (measured in the total amount of fat [kg] relative to height [in metres], visceral adipose tissue, android to gynoid ratio (based on the distribution of fat), fat free mass index, skeletal muscle mass (including the percent of the body that is composed to skeletal muscle), appendicular lean mass to height ratio (that measures amount of lean mass in arms and legs relative to height), and bone density (estimated density per unit area and relative to a defined population based on the Z-score which compares bone density of the individual to that expected in the normal population adjusted to age and body size and also the T- score that is relative to a healthy gender-matched population at peak bone mass age).

### Cardiovascular phenotyping

- Clinical chemistry. Blood (serum and plasma) will be sent for routine clinical chemistry including liver function test, kidney function test, full blood count, coagulation profile, lipid panel, blood glucose monitoring, uric acid, glycated haemoglobin. In addition, the following biomarkers will be collected: NT-ProBNP, GDF-15, Troponin I, hsCRP, IL6, D-dimer, ICAM, coagulation factors VIII, IX, X, XI and platelet reactivity measurement.
- Transthoracic echocardiogram (TTE) and vascular scans. All patients will undergo echocardiography, paying particular attention to global longitudinal strain and indices of stiffness and diastolic function. Endothelial assessments will include pulse-wave velocity, flow-mediated dilation (FMD), carotid and femoral intima-media thickness (IMT) assessments, ankle brachial index, and skin autofluorescence assessment for advanced glycation end products (AGE)^31^.
- CT coronary angiogram (CTCA) with coronary artery calcium score and epicardial fat assessment will be performed to delineate the coronary vasculature and epicardial fat.

### Liver phenotyping

- Transient elastography of the liver. Previous studies have shown the dose-dependent effect of hepatic steatosis and fibrosis in the development of coronary artery disease^32^, as well as in structural heart dysfunction in patients with Non-alcoholic fatty liver disease^33^. All patients will undergo vibration controlled transient elastography (VCTE) of the liver. This will provide quantitative surrogate measures of hepatic steatosis (via the controlled attenuation parameter, measured in dB/m), as well as non-invasive markers of hepatic fibrosis via the liver stiffness measurement (kPa). These will provide valuable information on associations and trajectories analysis relating extents of liver fat, inflammation and/or fibrosis to cardiometabolic phenotypes and CVD risk.
- Liver biopsy. NASH at presence remains a histological diagnosis. While non-invasive markers may predict the presence of fibrosis, there is yet to be a biomarker that reliably predicts NASH. As such, using the NAFLD cohort with biopsy proven and staged NAFLD, we will be able characterise the stage of NAFLD and/or fibrosis to evaluate presence of NASH and/or fibrosis vs NAFLD and its associated CVD risk.

### Molecular and multi-omics phenotyping

- Peripheral blood mononuclear cells (PBMC) will be isolated from whole blood samples. PBMC are separated from the plasma fraction of the blood samples by centrifugation. Detailed flow cytometry profiling of hundreds of distinct immune cell subsets will allow their enumeration, sorting and quantification based upon selected surface and signalling proteins. This can be blended with an immunometabolic profile analysis (based on cellular and plasma markers) and allow downstream “omic” analysis of cell subsets and single cells and immune repertoire mapping/generation of immune libraries. These cells would be banked and used for further genomic analysis, including whole genome sequencing, epigenetics, and transcriptomics analysis.
- Stool samples would be collected for the purpose of a microbiome analysis. The gut microbiome will be profiled at a community microbial ecology level (i.e. diversity). We will report alpha diversity, which measures the species richness and evenness within a single sample, using well-published methods such as Shannon’s or Simpson’s index. Then to compare the diversity between multiple groups or conditions, beta diversity analysis is performed, by employing distance metrics, such as Bray–Curtis or UniFrac, to assess similarities between microbial communities. We will also employ multivariate statistical methods like principal component analysis (PCA) to visualise and assess patterns in microbial community composition. Finally statistical tests such as analysis of similarities (ANOSIM) or permutational multivariate analysis of variance (PERMANOVA) will be employed to determine significant differences in microbial diversity between patients with and without CVD. Clusters of metagenomes associated with varying domains of metabolic health would be determined by the Dirichlet multinomial algorithms, whereby DMM bins, samples based on microbial community structure, and the approximate number of clusters is determined by the lowest Laplace approximation score. We will further internally validate the optimal number of clusters using an independent method (i.e. PAM clustering).

To then determine the specific taxonomic and functional features in the amongst patients in PICMAN associated with the varying domains of metabolic health, read counts will be transformed into relative abundances by normalisation to the total number of reads per sample.

c. Furthermore, exosome isolates, stool and urine samples will be banked for further multi-omics and validation analysis.
d. Patient-derived iPSCs will be differentiated into cardiac cell-types, such as cardiomyocytes, cardiac fibroblasts, and smooth muscle cells, and will undergo a battery of “omics” profiling covering their genomics, epigenomics, transcriptomics, and proteomics. We will also measure disease-relevant cellular phenotypes, which may include metabolic flexibility, contractility, electrophysiology, and morphology. Seahorse XF assays^34^ and single-cell RNA-sequencing^35^ will be used to measure the ability of iPS-derived cells to metabolise and switch between different substrates, and to plot trajectories between different metabolic gene programmes. Contractility and calcium handling can be evaluated through the IonOptix platform with cell shortening measurements and calcium-sensitive dyes. Patch-clamp^36^ techniques and multi-electrode arrays^37^ will be used complementarily to study electrophysiology either in bulk or in single cells. The morphology of iPS-derived cells will be examined through bright-field image analyses and immunofluorescence staining of various structural proteins. By combining whole-genome sequencing or genotyping information, genetic variation at specific loci can be linked to variation in multiple ‘omics' layers and cellular function through quantitative trait loci (QTL) analyses^38^. Colocalization of QTL and existing GWAS datasets will allow for the identification of putative targets driving disease aetiology.

### Neurovascular phenotyping

- Magnetic resonance imaging (MRI) and magnetic resonance angiogram (MRA) of the brain and intracranial vessels to assess for (1) ischemic lesions or white matter hyperintensities in the brain; (2) cerebral small vessel disease; (3) cerebral atrophy; and (4) intracranial atherosclerosis(including presence of plaque, degree of stenosis and plaque characteristics)^39^.
- Extracranial ultrasound doppler to evaluate (1) carotid intima-media thickness; (2) presence of plaque; and (3) extracranial vessels blood flow velocity as a measurement of degree of stenosis^40^
- Cognitive assessment Cognitive assessment will be performed using the Mini mental state exam (MMSE), the Montreal Cognitive Assessment (MOCA) and a digital cognitive battery of tests. The digital cognitive battery includes the Digital Processing Speed Test, Verbal Fluency Test and Trail Making Test^41^.

### Statistical calculation

This is an observational study to test feasibility. We plan to use PICMAN data for larger expanded cohorts and interventions and have not performed a sample size calculation for now. Amongst the cohort of individuals at elevated risk of ASCVD, the main outcome of interest is change in metabolic flexibility (ΔRQ). A case control comparison would be employed to assess differences in ΔRQ based on presence of non-alcoholic fatty liver disease, as well as to study the associations with cardiovascular measures of diastolic dysfunction and coronary and cerebral atherosclerosis, metabolic measures of insulin sensitivity, total body fat mass, volume of visceral adipose tissue and subcutaneous adipose tissue. Prior studies assessing metabolic flexibility have had smaller sample sizes, ranging between seven to twenty-five^42^.

Data will be analysed using descriptive statistics. As data will be non-normally distributed, we will present continuous variables as median and interquartile range and categorical variables as number (%). We will analyse data using the non-parametric Mann–Whitney *U*-test or Wilcoxon Rank Sum test for continuous variables and the chi-square test for categorical variables. Other statistical analysis will be conducted as required. Analysis will be performed using appropriate statistical software.

### Impact of the outcomes

The seriousness of metabolic disease and its burden in Singapore is rising. Fatty liver (NAFLD/NASH) represents a preceding or underlying condition, especially where it is hypothesised that quantifiable metabolic inflexibility may be representative of an individual in his/her silent, but high-risk progression towards insulin resistance and diabetes. Moreover, the undeniable cause of mortality and morbidity in patients with the wide spectrum of metabolic syndrome is CVD outcomes and events. Through deep and contemporaneous characterisation of the cardio-metabolic phenotype of a wide group of Singapore patients represented by PICMAN, we have an invaluable opportunity to converge the imaging, biochemical and metabolic biomarkers. We aim eventually to identify patients at the greatest risk of disease progression through novel means and uncover new disease biomarkers and targets for therapies.

### Ethics approval and consent to participate

Ethics approval was obtained under the National Healthcare Group Domain Specific Review Board, reference number 2021/00003, in June 2021. All methods were performed in accordance with the relevant guidelines and regulations.

## Discussion and plans

In Singapore, acute myocardial infarction afflicts patients in the 50 to 60 age group, which is 5–10 years younger than other high-income countries with the incidence of heart attacks rising from 7344 episodes in 2010 to 12,533 episodes in 2019^43^. This rise can be attributed, at least in part, to the increasing incidence of modifiable CV risk factors. As emphasised, missing risk factors among those who are seemingly well, are a growing concern. We see a clear need for data to provide material towards formulating focused implementation and adoption projects towards tackling this surging medical condition in Singapore.

Similarly, existing Asian heart failure (HF) cohorts have highlighted unique features of Asian HF patients when compared to HF patients in the USA and Europe. These include earlier age of onset, more aggressive disease with worse clinical outcomes, and unique HF clinical phenotypes (e.g. lean diabetic patients with heart failure with preserved ejection fraction)^44^.

Over the last decade in Singapore, there have been substantial increases in the prevalence of several major modifiable CVD risk factors including high blood pressure (36%), high blood cholesterol (39%), obesity (11%) and diabetes (10%), underscoring the national need to focus on the primary prevention of CVD. This is a long-neglected patient group with asymptomatic subclinical CVD who urgently need characterisation for drivers of disease. It is not known whether there are subgroups of phenotypes among Singaporeans with subclinical disease, or which subgroup(s) have precariously worse prognosis. One such example is individuals without standard modifiable risk factors, who may have a worse prognosis after acute coronary syndrome^45^.

We hypothesise that early CVD is sub-classifiable by inter-related myocardial-vascular-liver-metabolic-lifestyle-molecular phenotypes. This study is the first deeply-phenotyped early subclinical CVD cohort in Singapore, which will then serve as a foundational platform for biomarker and target discovery. The substantial and unique preliminary dataset acquired will enable us to proceed to larger prospective observational studies as well as future interventional studies.

In Asian populations there is also a high rate of intracranial stenosis, which is not fully explained by the incidence of diabetes or smoking^46^. It is often cited that Caucasian populations have a higher rate of extracranial atherosclerotic disease, however, Asians can also have a high incidence of concurrent extracranial and intracranial stenosis^47^. The causes behind these stenoses are largely unknown and this study will help elucidate parts of it.

In particular, we aim to investigate the role of metabolic inflexibility as an early marker in the pathogenesis of cardiometabolic disease. Metabolic inflexibility is involved in the pathophysiology of insulin resistance, the predominant factor linked to type 2 diabetes [4]. It has also been associated with obesity and abnormal adipose tissue and skeletal tissue metabolism. This offers the most direct link to the metabolic syndrome, associated with the development of cardiovascular disease. Conceptually, if detected early enough before the development of irreversible cardiometabolic disease, this may serve as a useful biomarker to allow for screening of at-risk individuals as well as a therapeutic target.

Metabolic flexibility is calculated by a change in respiratory quotient (ΔRQ) as measured by the difference in RQ between the fasting state and after a liquid mixed meal challenge. Importantly, this shift in cellular fuel selection from a predominantly oxidative fatty acid metabolism to glucose oxidation (and vice versa) that characterises healthy metabolic flexibility during the transition in energy demands (e.g. from fasting to fed states or from resting to exercise) is a process driven by insulin and the latter’s action on enzymatic activity in the biochemical pathways of glycolysis, gluconeogenesis, ketogenesis, fatty acid biosynthesis and anaplerosis of the Kreb’s cycle mediated by metabolites fluxes between the cytosol and the mitochondrial matrix. This delicate balance in fuel substrate shift is governed by the interaction between AMPK (master cellular energy sensor) and mTOR (master cellular nutrient sensor) at the molecular level. Notably, AMPK which is activated by fasting and exercise, promotes catabolism by upregulating fuel oxidation for energy release, whereas mTOR which promotes anabolism by suppressing mitochondrial biogenesis by contrast is activated by feeding and insulin, and inhibited by AMPK itself. Given the tight coupling between metabolic flexibility and insulin action, it is readily conceivable why metabolic flexibility correlates to insulin resistance. Moreover, metabolic flexibility or inflexibility not only correlates with insulin resistance, it also reflects the physiological impact of insulin resistance on cellular energy homeostasis. Hence, while tools such as the hyperinsulinemic euglycemic glucose clamp and intravenous glucose tolerance test may assess the insulin sensitivity with great precision, these tests by themselves only evaluate glucose uptake but do not show the extent of impact on other aspects of metabolic physiology unlike the information that comes from the evaluation of metabolic flexibility. The measurement of metabolic flexibility therefore offers additional insights into the derangement of metabolism beyond what insulin resistance can provide. Furthermore, unlike the labour-intensive clamp and IVGTT experiments, metabolic flexibility is easily measured with good reproducibility using indirect calorimeter mounted on a portable metabolic cart which is readily operated by a single clinician or a research personnel at a fraction of the cost of either insulin sensitivity methods above. Such aspects of metabolic flexibility coupled with its lower costs justifies our choice for its usage in this present PICMAN study.

In Phase II, we will set out to assemble a larger cohort that leverages stronger statistical calculations, especially for longitudinal follow up for outcomes.

In Phase III, we will target therapeutic interventions such as intermittent fasting, ketogenic diet, lifestyle interventions or cardiovascular therapies with recently demonstrated successes: SGLT-2 inhibitors, and the anti-inflammatory colchicine, to assess their effect on metabolic flexibility and cardiometabolic function.

### Limitations

PICMAN is a proof-of-concept study sponsored by an intramural institutional research grant. There is a rigorous protocol of investigations to study the myocardial-vascular-liver-metabolic-lifestyle-molecular phenotypes and lay the foundation for target discovery in early CVD. Due to the resources required, one limitation is the sample size possible with the current resources. Even so, given the depth of the metabolic phenotyping with the underlying aim of characterising metabolic flexibility, this is still considered a large study compared to previous ones^42^. However, as a preventive cardiology cohort, the sample size is small and will not allow for definitive conclusions. To bridge this gap, we intend to make use of substratification methods for cardiometabolic risk, making use of established as well as newly-developed methods^48, 49^. In addition, we hope to build upon this study to embark on future larger scale studies. Furthermore, resource limitation only allows us to phenotype this cohort at a single time-point. Efforts are underway to obtain further funding to allow future longitudinal phenotyping and interventional studies.

A further limitation is that some tests performed, such as whole-body MRI for body composition profiling or metabolic assessments such as indirect hood calorimetry, are expensive and are not immediately implementable in clinical care pathways. Complementary and more readily-available tests such as DXA scans for assessment of fat distribution have been included to allow for the study of correlation and implementation in a clinical context.

## Summary

The PICMAN study seeks to lay the foundations for further future larger scale and more experimentally ambitious studies based on the overarching hypothesis that metabolic flexibility is an early marker in subclinical cardiovascular disease, indicating a point of timely intervention where the disease process is still reversible. The importance of a deeply-phenotyped local cohort will allow us to study the progression of CVD in seemingly well adults, converging imaging, biochemical, metabolic markers, and ‘omics' analysis.

## Data Availability

Upon completion of the study, data and materials would be made available upon request. For data requests, kindly contact the Principal Investigators, Dr Mayank Dalakoti or Prof Roger Foo.
